# Supplementary material for: Disparity of cycad leaves dispels the living fossil metaphor
Source: Commun Biol. 2024 Mar 14;7:328. doi: 10.1038/s42003-024-06024-9 (PMC10940627; doi:10.1038/s42003-024-06024-9)
Supplement: Supplementary file 2 — Supplementary Material [file 42003_2024_6024_MOESM2_ESM.pdf]

Supplementary Methods S1: New characters

Character 1: Lamina attachment: (0) adaxial, (1) medial. From character 26 of Martinez et al. (2012).
Character 2: Leaf architecture (0) Once pinnate (1) Bipinnate (2) Taeniopteroid. Recodified from character 24 of Martinez et al. (2012).
Character 3: Leaflets (0) regular (1) irregular. This character was erected to distinguish the irregular, dissected leaflets present in Nilssonia from the more regular leaflets of other cycads.
Character 4: Leaflet insertion (0) decurrent (1) articulate. From character 27 of Martinez et al. (2012). Character 5: Leaflet margin (0) entire (1) dentate (2) serrate. This character was erected to distinguish the different leaflet margins, which are commonly used to distinguish between genera (i.e. Dioonopsis, Encephalartites). We distinguish the non-vascularized teeth present in Dioon and Encephalartos from the vascularized teeth present in Bowenia and Zamia.
Character 6: Leaflet venation (0) Parallel veins (1) Single vein. Recodified from character 30 of Martinez et al. (2012).
Character 7: H-anatomoses (0) Absent (1) Present. This character was erected to distinguish the situation present in *Ctenis* and other fossil leaves, where the anastomoses between veins do not correspond to reduction in vein numbers.
Character 8: terminal anastomoses (0) absent (1) present. Recodified from character 33 of Martinez et al. (2012).
Character 9: Midvein in the leaflets (0) Present (1) Absent. This character distinguishes the multiveined leaves of *Stangeria* or *Zamia* (*Chigua*) restrepoi, which present a midrib, from all other multiveined leaves.
Character 10: Leaflet midvein type: (0) Cycas-type (1) Stangeria-type. Modified from character 32 of Martinez et al. (2012).
Character 11: Stomata (0) flush (1) sunken. Character 50 of Martinez et al. (2012).
Character 12: Stomatal orientation (0) longitudinal (1) random. Modified from character 52 of Martinez et al. (2012).
Character 13: Stomatal disposition (0) In bands between veins (1) diffuse (2) in groups. This distinguishes between taxa with no clear stomatal bands, taxa with stomatal bands and taxa with clumps of stomata that do not correspond to intervein bands (such as Ctenis minuta).
Character 14: Coronal rim (0) absent (1) present. This structure is similar to the accessory cell corona of Martinez et al. (2012), i.e. a cuticular rim that surrounds the stomatal opening. It is typical of many Ctenis taxa.
Character 15: Substomatal complex (0) non thickened (1) thickened. Character 89 from Coiro and Pott (2017)
Character 16: Subsidiary cells (0) thick cuticle (1) thin cuticle. This character distinguishes the Zamiaceae from other cycads.
Character 17: Stomatal pit (0) absent (1) present. This character indicates the presence of a chamber formed by the encircling cells (like in Dioon or Cycas revoluta).
Character 18: Lateral encircling cells (0) zero (1) one (2) two (3) three. Modified from character 53 of Martinez et al. (2012).
Character 19: Papillae on the stomatal pit (0) absent (1) present.
Character 20: Polar encircling cells (0) absent (1) present. This character indicates the presence of differentiated polar encircling cells.
Character 21: Polar encircling cells overarching (0) absent (1) present. This character indicates whether the polar encircling cells elongate to overarch the poles of the guard cells.
Character 22: Polar cuticular extension (0) absent (1) present. This character describes the presence of a cuticular intrusion between the poles of the guard cells and the polar pavement cells.
Character 23: Anticlinal pegs (0) absent (1) present. Character 48 of Martinez et al. (2012).
Character 24: Epidermal cells in stomatal bands (0) elongated (1) isodiametrical. Elongated cells are typical of most extant Zamiaceae.
Character 25: Thin-walled cells (0) absent (1) present. Modified from character 46 of Martinez et al. (2012).

Character 26: Thin-walled cell files (0) on the costal epidermis (1) on the intercostal epidermis (2) absent. This character separates the typical cells files of Ceratozamia and Dioon.
Character 27: Anticlinal walls (0) straight (1) wavy.
Character 28: Trichome bases (0) circular (1) angular. Angular trichome bases are typical of Cycas and other fossil genera.
Character 29: Cuticular striae (0) absent (1) present. Modified from character 47 of Martinez et al. (2012).
Character 30: Epidermal idioblasts (0) absent (1) present. Epidermal idioblasts are typical of some species of Dioon and Macrozamia.
Character 31: Stomatal distribution (0) hypostomatic (1) amphistomatic. Character 49 of Martinez et al. (2012).
Character 32: Basiscopic leaflet margin: straight (0); concave (1); convex (2).
Character 33: Acroscopic leaflet margin: straight (0); concave (1); convex (2).
Character 34: Basiscopic spines: Absent (0); Present (1).
Character 35: Acroscopic spines: Absent (0); Present (1).
Character 36: Basiscopic leaflet base: Decurrent (0); “non-decurrent” (1). Non-decurrent here refers to both the articulate base of the Zaminae and a non-articulate leaf base that does not decurr across the rachis.
Character 37: Acroscopic leaflet base: Decurrent (0); “non-decurrent” (1).
Character 38: Basiscopic leaflet base angle: right (0); acute >45 degrees (1); acute <45 degrees (2); obtuse (3).
Character 39: Acroscopic leaflet base angle: right (0); acute >45 degrees (1); acute <45 degrees (2); obtuse (3).
Character 40: Leaflet apex: Acute (0); Rounded (1).
Character 41: Leaflet attachment: As broad as middle of leaflet (0); broader than middle of leaflet (1); narrower than the middle of the leaflet (2).
Character 42: Leaflet symmetry: Symmetric (0); Asymmetric (1).
Character 43: Leaflet morphology across the leaf: Uniform (0); Heterogeneous (1).
Character 44: Angle of the medial leaflet on rachis: Right (0); Acute (1).
Character 45: Bifurcated leaflets: Absent (0); Present (1).

Supplementary Figure 1

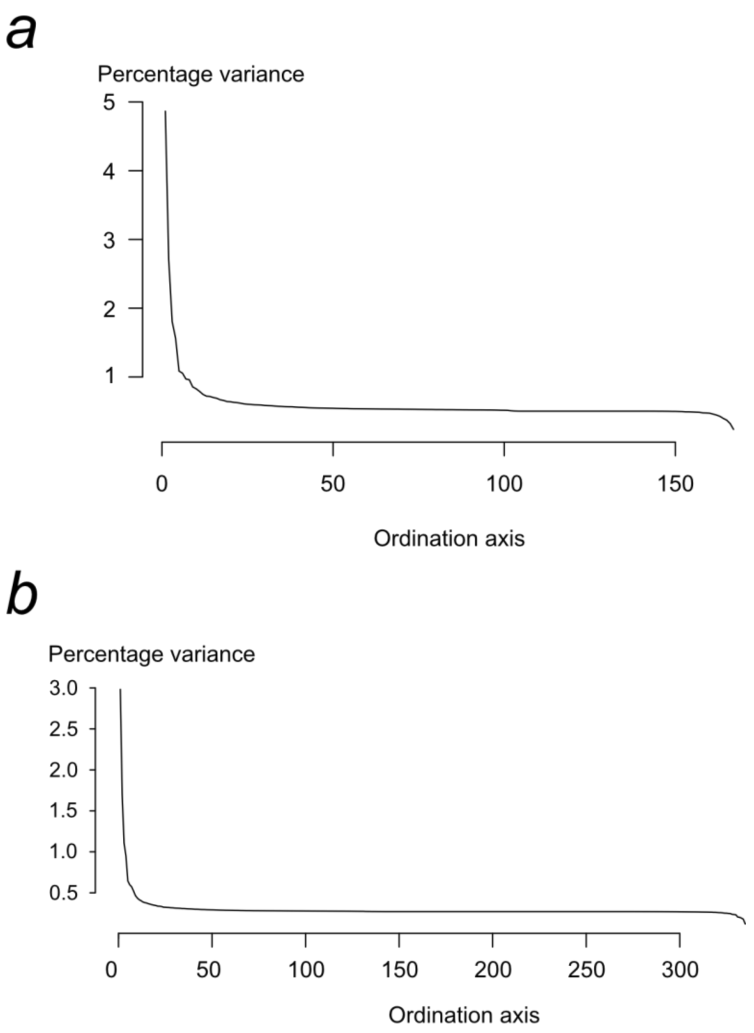

Supplementary Figure 1: Scree plot indicating the variance explained by each Principal Coordinate analysis axis for the Only Taxa morphospace (a) and the Pre-Ordination Ancestral State Reconstruction morphospace (b).

Supplementary Figure 2

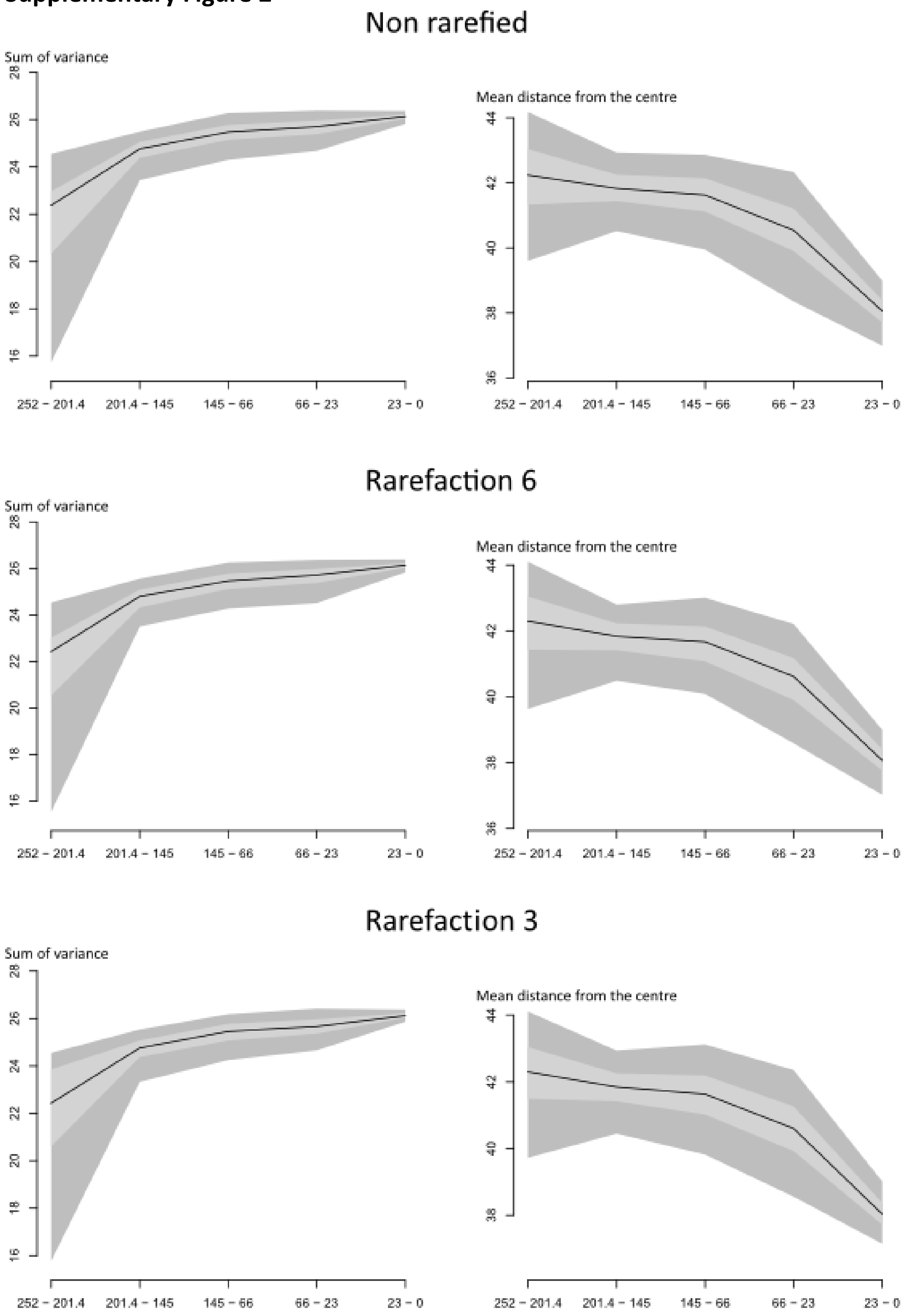

Supplementary Figure 2: Disparity Through Time graphs for the OT morphospace showing the effect of rarefaction. Sum of Variance (right) and Mean Distance from the Centre (left) are shown for Non-rarefied datasets (top), datasets rarefied to six data points (middle) and datasets rarefied to three data points (bottom).

Supplementary Figure 3

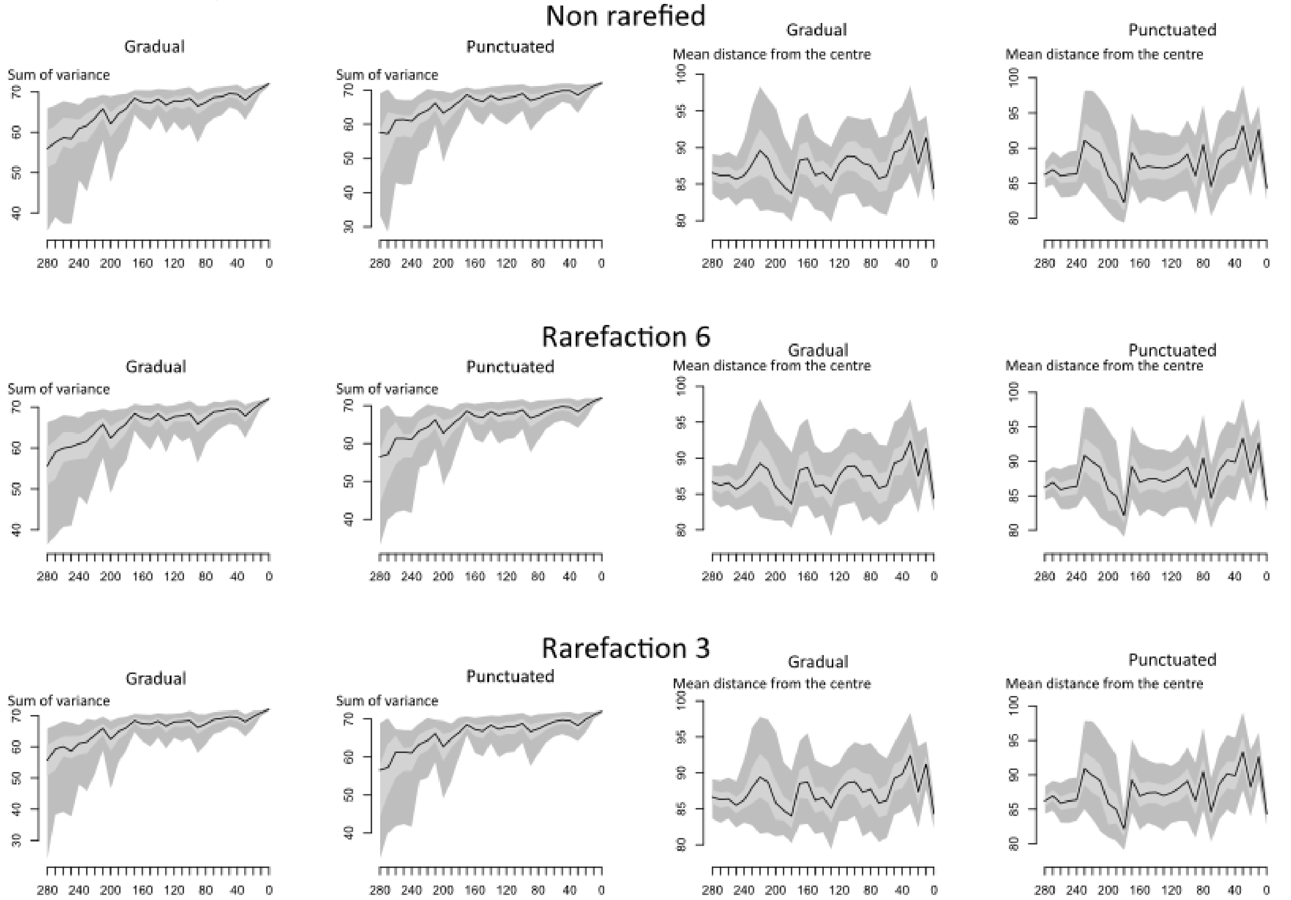

Supplementary Figure 3: Disparity Through Time graphs for the Pre-Ordination Ancestral State Reconstruction morphospace showing the effect of rarefaction. Sum of Variance (two columns on the right) and Mean Distance from the Centre (two columns on the left) are shown for Non-rarefied datasets (top), datasets rarefied to six data points (middle) and datasets rarefied to three data points (bottom). Both gradual and punctuated models of disparity reconstructions are shown.

Supplementary Figure 4

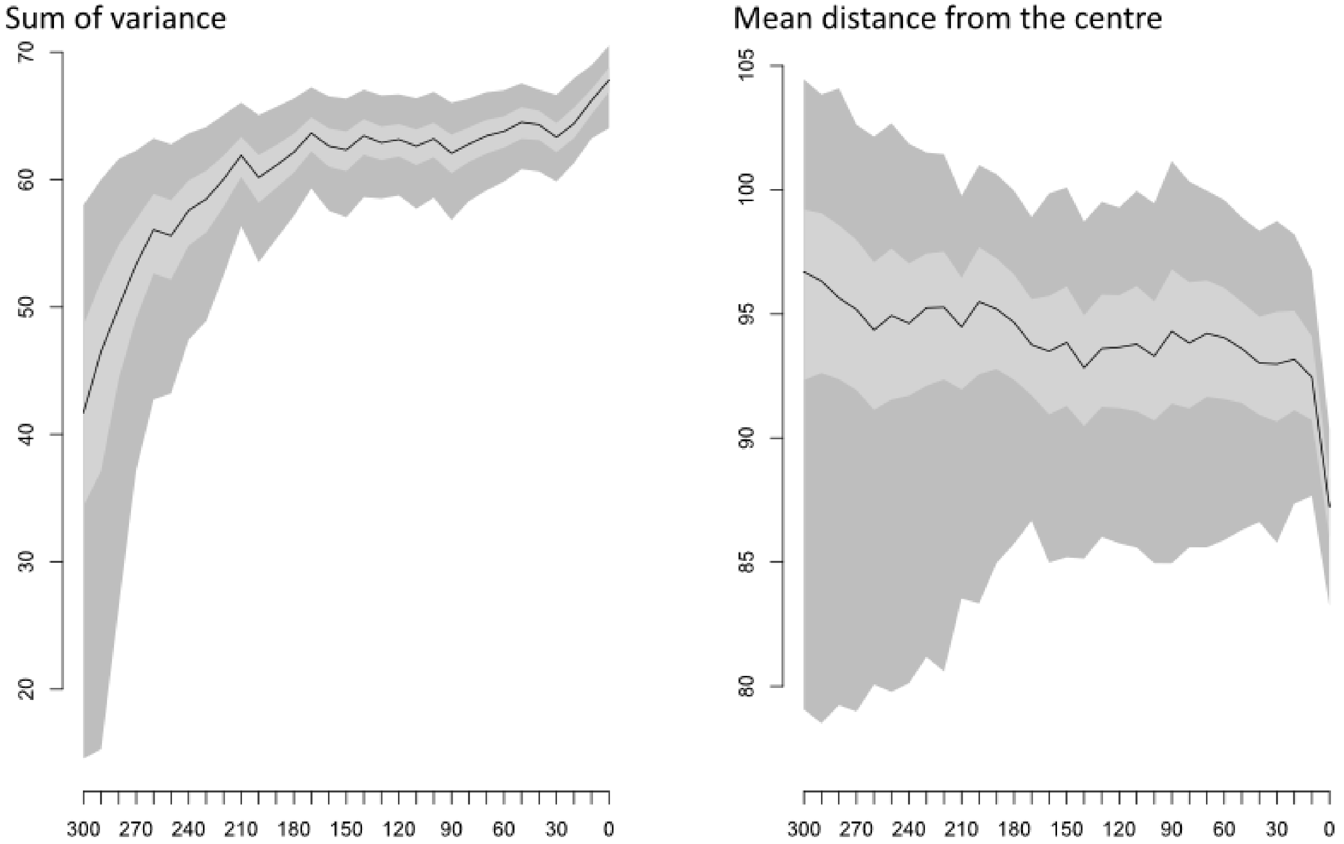

Supplementary Figure 4: Disparity Through Time graphs for the Pre-Ordination Ancestral State Reconstruction morphospaces using the gradual model reconstructed over 100 random trees from the posterior analysis of [17], showing the robustness of the results to topological uncertainty.

Supplementary Figure 5

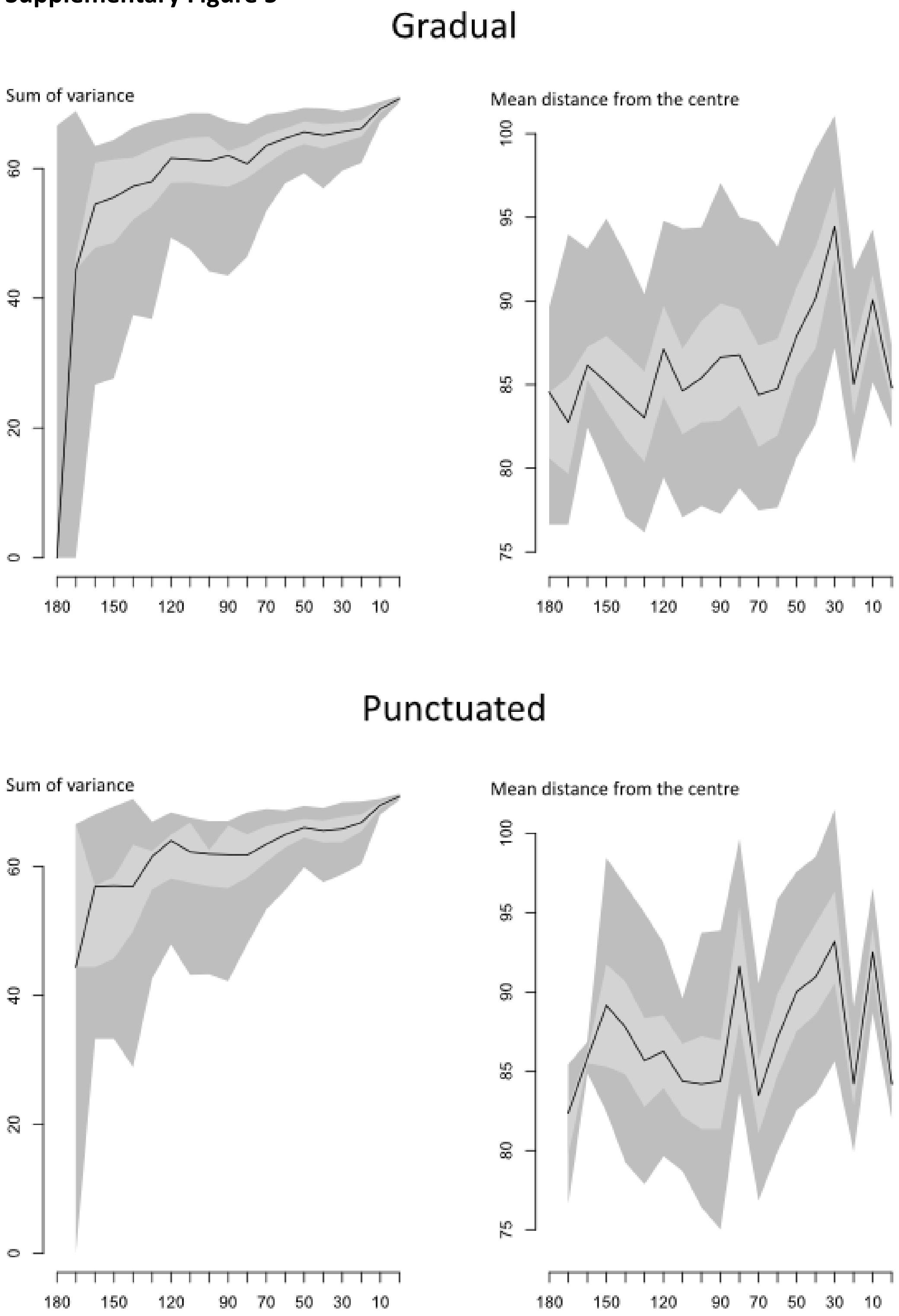

Supplementary Figure 5: Disparity Through Time graphs for the Pre-Ordination Ancestral State Reconstruction morphospace for the Zamiaceae. Sum of Variance (right) and Mean Distance from the Centre (left) are shown for both the gradual (top) and punctuated (bottom) model of disparity reconstruction.

Supplementary Figure 6

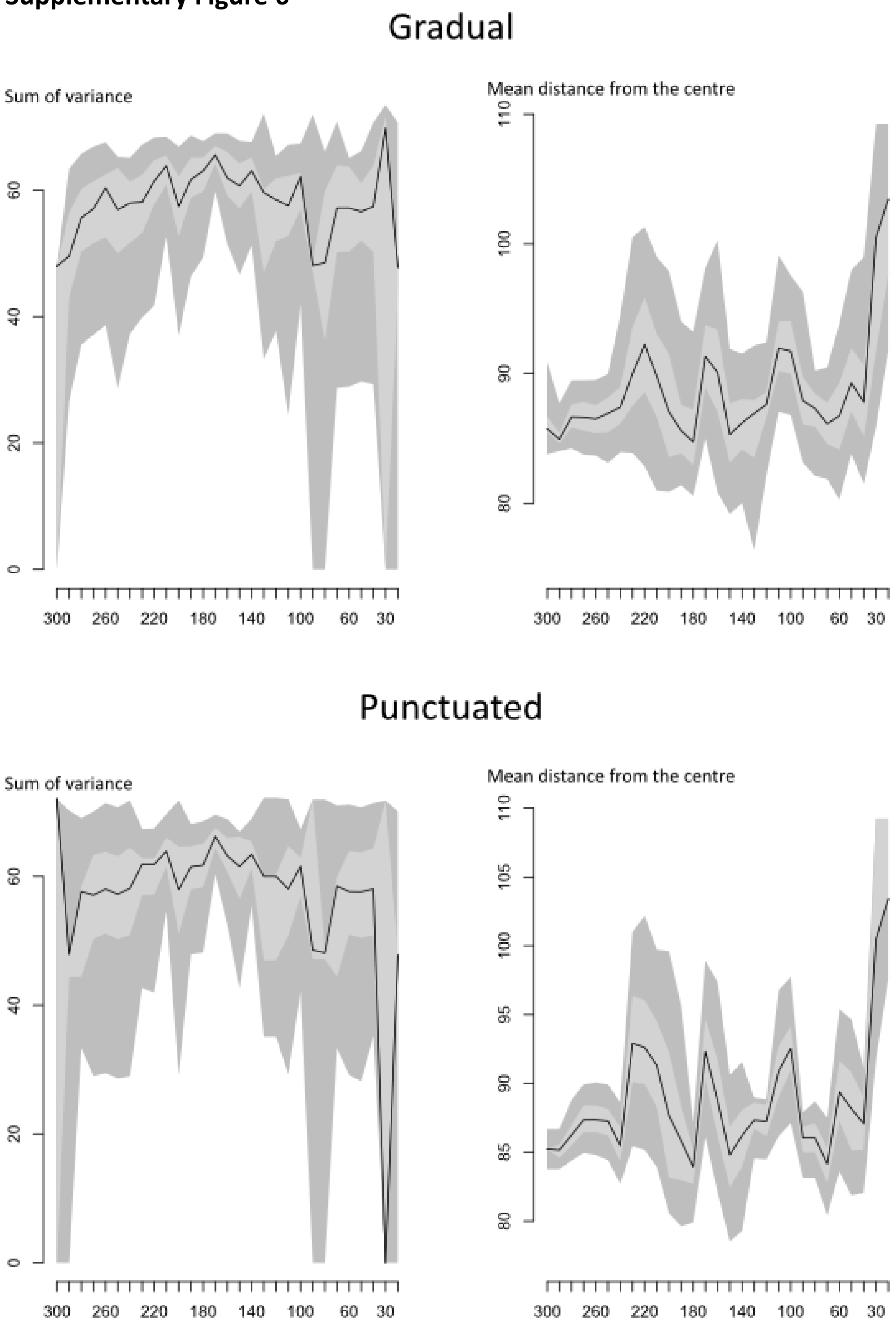

Supplementary Figure 6: Disparity Through Time graphs for the Pre-Ordination Ancestral State Reconstruction morphospace for the extinct cycads. Sum of Variance (right) and Mean Distance from the Centre (left) are shown for both the gradual (top) and punctuated (bottom) model of disparity reconstruction.

Supplementary Figure 7

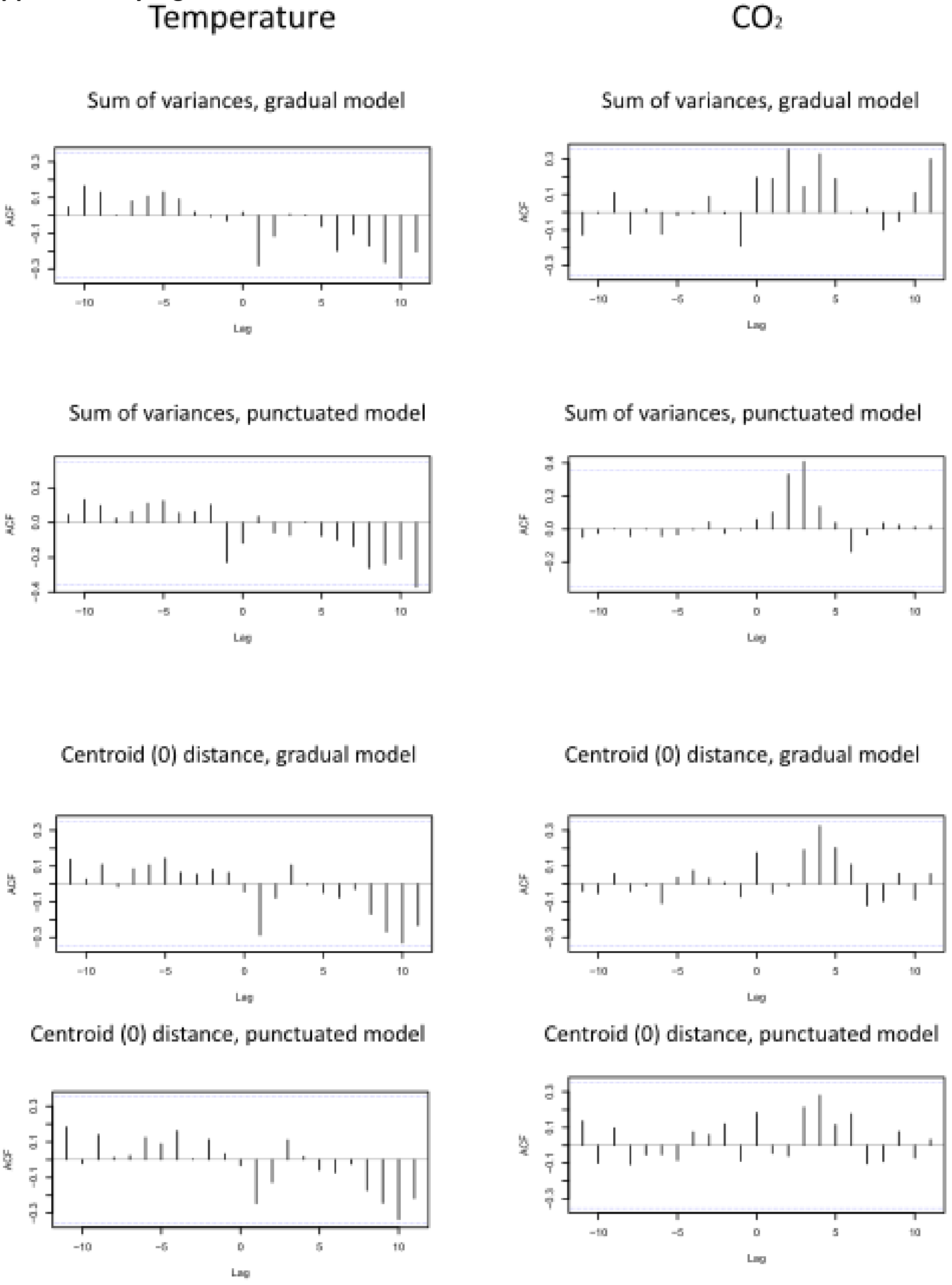

Supplementary Figure 7: Results of the cross-correlation analyses between the residuals of the Autoregressive Integrated Moving Average (ARIMA) model for Temperature (left) and CO_2 (right) and the residuals for the bootstrapped median of different disparity metrics and reconstruction methods from the same ARIMA model. The graphs show that all correlation coefficients are lower than 0.5, indicating weak correlation across all disparity metrics and predictors.
